# The Influence of the Affinity between Aggregate and Bitumen on the Mechanical Performance Properties of Asphalt Mixtures

**DOI:** 10.3390/ma14216452

**Published:** 2021-10-27

**Authors:** Maria M. A. S. Maia, Marisa Dinis-Almeida, Fernando C. G. Martinho

**Affiliations:** 1 Centre of Materials and Building Technologies (C-MADE), University of Beira Interior, Edifício II das Engenharias, Calçada Fonte do Lameiro, 6200-358 Covilhã, Portugal; maria_msa5@hotmail.com; 2 CERENA, Instituto Superior Técnico, Lisbon University, 1049-001 Lisbon, Portugal; fernando.martinho@tecnico.ulisboa.pt

**Keywords:** adhesion promoter, affinity aggregate–bitumen, filler, permanent deformation, water sensitivity

## Abstract

Two of the main problems encountered in flexible pavements are the stripping of coarse aggregates and the formation of rut depth due to increases in the volume of road traffic and heavy vehicle loads, especially in areas where speeds are low. The existence of rut depth also affects the comfort and safety of road users due to the water accumulation on the pavement surface and reducing tire/pavement friction, which can lead to hydroplaning phenomena. In this research, it was proven that the use of fillers of different origins influences the affinity between aggregates and the binder. The effect of an adhesion promoter in the mix design (such as the amine included in cellulosic fiber pellets) was also studied. Several tests were carried out to determine the binder/aggregate adhesiveness, water sensitivity and resistance to permanent deformation, to evaluate the performance of different blends. It was found that the addition of this additive increased 10% of the aggregate surfaces covered with bitumen when compared with the aggregates without this addition. As expected, the water sensitivity tests showed that the mixture with granitic filler had the lowest indirect tensile strength ratio (ITSR) value (70%), while the mixtures with limestone filler led to the highest percentages (ranging from 83 to 93%). As for the results of the wheel tracking tests (WTT), it was confirmed that the use of limestone filler translates into an improvement in the performance against the permanent deformation of the asphalt mixtures. The mixture with higher bitumen content and adhesion promoter revealed the best average results.

## 1. Introduction

Over the past few decades, road administrations around the world have become aware of the importance of extending the life cycle of asphalt mixtures included in different layers of road pavements. The need for an increase in the durability of these materials is important and urgent for the reduction in natural resources that are spent on their maintenance and rehabilitation. One way to intervene in this regard is to seek greater adaptation of the type of bituminous mixture (and its constituents), taking into account the characteristics of the works and materials available in each region. For example, where there is a great availability of granitic aggregates, it is essential to study the influence of their affinity with different binders. The study of resistance to permanent deformation, as well as the water sensitivity of these bituminous mixtures, are two of the other parameters that deserve to be well evaluated.

As explained by Zhang et al. [1], bitumen is usually acidic so, when mixed with acid aggregates, some repulsion interactions occur which promote partial bonds where there are only a few hydrogen ions. The stripping phenomenon in asphalt mixtures is one of the most important types of distress in flexible pavements [2]. Usually, the affinity between aggregate and bitumen is described by four theories, isolated or combined [3]: molecular arrangement, mechanical adhesion, surface energy and chemical reaction. Several factors affect these theories, namely: chemical composition of materials; viscosity of bitumen; texture, angularity, shape, porosity and cleaning of aggregates; surface tension in the interface of bitumen–aggregates; and temperature in the mixing or the presence of moisture.

As for the texture, angularity and shape of aggregates, Wang et al. [4] showed that for three types of aggregates, through a simplex lattice design (SLD) method, their morphological characteristics are correlated with the viscoelastic properties of bituminous mixtures (at high temperature) and improves rutting resistance.

Regarding the manufacturing/application temperatures of bituminous mixtures, it is known that some additives used in warm mix asphalt (WMA), and in certain percentages, contribute to the reduction of water damage, such as that concluded by, for example, Kassem et al. [5].

Many fillers [6,7,8,9] and special additives (such as silane, amine, rubbery polymers [10] or other nanomaterials—nanoclays [11]), have also been used to enhance the adhesion between binders and acidic aggregates (that tend to be hydrophilic [12]). For example, chemical or anti-stripping additives have generally improved the adsorption interface of aggregates–bitumen and reduced the binder debonding (due to moisture) from the surface of the aggregates [13].

Although Aguiar-Moya et al. [3] stated that an increase in aggregate-binder strength may not improve the water-resistance of the bituminous mixtures, other authors, such as Hamedi et al. [12], Cui et al. [14] and Lucas Júnior et al. [15], reported that a better water-resistance and fatigue life can be achieved by introducing adhesion promoters in their compositions. Cui et al. [14] tested the use of two different silanes and an anti-stripping amine. Among other conclusions, they found that the amine-based adhesion promoter was very effective on blends that included unmodified bitumen and granitic aggregates. Ding et al. [16] used another nanomaterial, having verified that a silane coupling agent (SCA) also improved the adhesion between granitic aggregates and bitumen. Other authors have comprehensively studied the beneficial effect of adding special adhesion promoters on asphalt mixtures, as was the case of Liu et al. [17], who used a plant ash by-product mixed with two bitumen grades and three types of aggregate (including one granite).

Zhang et al. [18] investigated the influence of the mineralogical composition of four aggregates (two of which are granitoid) and two unmodified binders on the water-resistance of the aggregate–bitumen bond. They observed a good correlation between the mineral composition of the aggregate and moisture absorption, as well as a greater influence of the aggregate geological nature (than the type of binder) in the sensitivity to moisture. Yin et al. [19] studied the influence of the chemical compositions of some aggregates on the quality of the bitumen–aggregate interface, having proved (in the case of granite) that this link is oriented only by its physical adhesion with the binder. In another study, Feng et al. [20] also evaluated the mineral compositions of different aggregates, in addition to their surface texture, having concluded that these properties have a significant impact on the behavior of the interface between bitumen and coarse aggregates.

In a recently published study [21], the researchers used a rolling bottle test (RBT) and molecular dynamics simulation (MDS) to prove that aggregates with higher content of chlorite, nepheline, olivine and pyroxene minerals will affect water sensitivity of the bituminous mixtures less than those that include higher content of plagioclase, quartz and calcite. On the other hand, Cong et al. [22] demonstrated that the asphalt binder fractions (asphaltenes, resins, aromatics and saturates) had a greater influence on moisture sensitivity than the composition of the aggregate. However, Liu et al. [23] have proven that the physical–chemical properties of aggregates may make a greater contribution to moisture damage than bitumen properties. Meanwhile, Cui et al. [14] also stated that the porosity of the aggregates was less important than their chemical composition.

In summary, as concluded by Zhang et al. [24], the mechanical properties of the interface between binder and aggregates depends on several aspects, both elastic and viscous (plastic) regions are found at the aggregate/binder interface when tensioning a sample. The geological nature of the aggregate and the aging have a significant effect on the tensile strength of this interface. Aging also favors the removal of the binder from the aggregates (stripping), but if these include Al_2_O_3_ this problem can be mitigated. The nature of the aggregate, and the time and degree of aging, greatly affect the mechanisms and adhesive properties of the binder to the aggregate.

Many advanced tests and simulations can be performed to evaluate the quality of the binder-aggregate bonding, such as X-ray photoelectron spectroscopy (XPS) [19,25]; energy dispersive X-ray (EDX) [25]; active adhesion evaluation method (AAEM) [10]; atomic force microscopy (AFM) [26,27]; binder bond strength (BBS) [28]; scanning electron microscopy (SEM) with energy disperse spectroscopy (EDS) [17]; infrared spectroscopy (IR) [13]; optical microscopy (OM) [29], hyperspectral imaging/digital image processing (DIP) [30] and molecular dynamics simulation (MDS) [16,21,24]; in addition to other test methods described by Mehrara et al. [2].

Some “traditional” tests [2], such as wheel tracking (one of twelve rutting performance indicators listed in [31]), can also be used, not only to assess the permanent deformation resistance of bituminous mixtures, but also to evaluate their moisture sensitivity [2,32]. For instance, Han et al. [33] tested several hot-mix asphalt mixtures in a Hamburg Wheel Tracker (HWT), having concluded that this device offers a good correlation with field performance. At the same time, they also observed an enhancement in the moisture sensitivity when using anti-stripping additives.

The hypothesis of increasing the durability of surface layers, resulting from a better affinity between a specific acid aggregate (coarse-grained granite) and an unmodified binder (by changing the nature of the filler or adding a specific chemical compound), motivated the research described in this manuscript. Thus, the main objective of this study was to assess the changes to certain properties of some traditional bituminous mixtures caused by these modifications.

Four mixtures were tested, all of which just included granitic aggregates. The experimental program was then started with the mix design. After selecting the optimum bitumen content (through the Marshall method, under the European standard EN 12697-34 [34]), the influence of the addition of limestone filler and adhesion promoter were analyzed. The affinity between aggregate and bitumen was also verified, and the mechanical performance of the studied mixtures was assessed through water sensitivity and permanent deformation resistance tests.

This paper describes in some detail all materials used in the aforementioned research; the samples preparation; the test equipment, its configuration and procedures; as well as the results obtained and some conclusions that can be inferred.

## 2. Materials and Methods

### 2.1. Materials

#### 2.1.1. Binder

An unmodified 50/70 penetration grade bitumen was used in the production of selected asphalt mixtures suitable for application in surface layers and colder regions. The temperature used in the production was defined in the range between 150 and 160 °C and the compaction of the specimens was achieved at (145 ± 5) °C. This neat binder had a penetration value of 59 mm × 0.1 mm (EN 1426 standard [35] @ 25 °C, with 100 g, during 5 s) and a softening point ring and ball (t_R&B_) of 47 °C (EN 1427 [36]).

#### 2.1.2. Aggregates and Fillers

**Figure 1:**
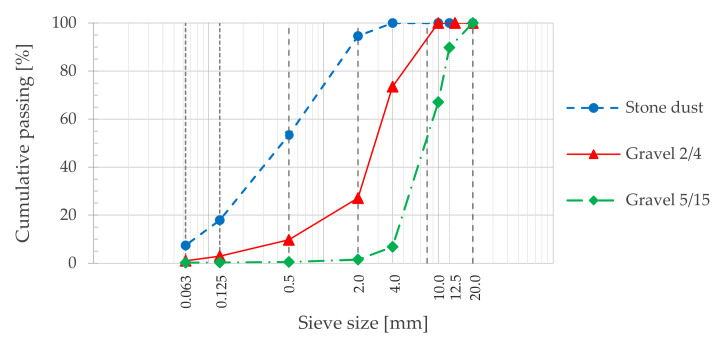
Granitic aggregates grading curves.

Three different fractions of the same granitic crushed rock were used (stone dust, 2/4 and 5/15 mm gravels), as well as granitic and limestone fillers (the first one was recycled from the asphalt plant and the second was a hydraulic lime). The grading curves of the granitic fractions, defined according to the European Standard EN 13108-1 [37], are shown in Figure 1.

Some of the aggregates and fillers properties are presented in Table 1. These values were obtained in this research and within the scope of the suppliers’ FPC (factory production control).

#### 2.1.3. Adhesion Promoter

In order to improve the behavior of the bituminous mixtures, increasing their durability and performance, cellulosic fiber pellets with an adhesion promoter were added to one of the compositions. This additive is composed of a mixture of natural cellulose fibers [38], bitumen and a specific amine. As claimed by its producer, these pellets of cellulosic fibers (which act as a carrier of the adhesion promoter) comprise several advantages, such as an aging reduction and improvement in the long-term performance of the bituminous mixtures (as a result of a higher affinity between aggregates and bitumen).

#### 2.1.4. Bituminous Mixtures

Four traditional bituminous mixtures were produced and tested. The first three blends were of the *AC 14 surf* type (asphalt concrete), a traditional mixture used in Europe as the surface layer, with a nominal maximum particle size of the aggregates of 14 mm. One of these mixtures included a granitic filler (GF), AC 14 *GF*, and the other two included a limestone filler (LF), AC 14 *LF1* & AC 14 *LF2*, in different percentages. The fourth mixture was a stone mastic asphalt (SMA) with an upper sieve size of the aggregate of 11 mm, also for surface course (*SMA 11 surf* type), which included an adhesion promoter (AP), SMA 11 *AP*, and was characterized by a discontinuous aggregate mixture, having a higher amount of binder. These mixtures were designed according to the Portuguese (European) standard [37] and Spanish specifications [39] (for SMA 11) and their compositions are presented in Table 2.

**Figure 2:**
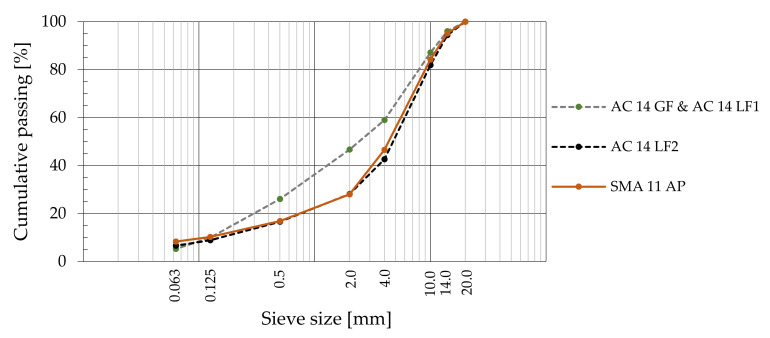
Grading curves were adopted for each bituminous mixture.

The AC 14 *LF2* and SMA 11 *AP* bituminous mixtures had similar granulometric curves. These and the other two grading curves adopted for the studied mixtures are presented in Figure 2.

After compaction of specimens (using the European standards EN 12697-30 [40] for cylindrical specimens and EN 12697-32 [41] for slabs), the studied bituminous mixtures were submitted to the mechanical performance assessment tests described in the next section.

The samples taken from each of the four blends (loose mixtures and cylindrical specimens) presented the volumetric properties shown in Table 3.

### 2.2. Methods

#### 2.2.1. Affinity Aggregate/Bitumen Test

Different tests can be performed to assess the affinity between aggregate and bitumen, namely those specified in the European Standard EN 12697-11 [42] (rolling bottle method, static method and boiling water stripping method). These tests can also be used as a supporting tool during the mix design, helping to find a binder with greater affinity to a given aggregate or vice versa.

In this research, a dynamic method with a rotating bottle with water (through the visual record of bitumen covering the aggregate) was the chosen test to assess this parameter. This test includes the preparation of 600 g of an 8/11 mm fraction of the aggregate, mixed with 16 g of bitumen (≈3 wt.% on mixture) and adhesion promoter (in the second case). The mixture is then divided into 3 parts, each of which is transferred to bottles that, posteriorly, will be filled with water.

The test begins with the placement of the bottles in the rolling machine (CONTROLS- model 75-B0011/A, Liscate, Italy), working at a speed of 60 rpm. After 6 h ± 15 min at room temperature, between 15 and 25 °C, the first reading is done. The affinity is expressed by the visual record of the aggregate surface covered with bitumen (by two different technicians) after the influence of this mechanical stirring (as a percentage of the total surface of the aggregate). After this first period, the degree of bitumen coverage of the particles is estimated, and the test continues for up to 24 h. In the end, the percentage of the aggregate surface still covered with bitumen is measured again and a graph is drawn with the average results. Due to the difficulty in obtaining a rigorous visual assessment by the technicians, this method involves some uncertainty resulting from their subjectivity.

#### 2.2.2. Water Sensitivity Test

The water sensitivity test was performed according to the European standard EN 12697-12:2008 [43]. For each optimum bitumen content, six cylindrical specimens were molded with a lower number of blows (2 × 50) than that used in Marshall specimens (2 × 75). These specimens were separated into two groups: one was maintained in the air at (20 ± 5) °C for a period of (72 ± 2) h (dry group) and the other (wet group) was previously subjected to vacuum in water for 30 min under an absolute pressure of (6.7 ± 0.3) kPa, followed by water bath at (40 ± 2) °C during the same period of (72 ± 2) h. Then, these two groups were tested (in a compression testing machine—UBI, Covilhã, Portugal) for indirect tensile strength, at (25 ± 2) °C, respecting the EN 12697-23:2003 [44] standard (indirect tensile strength, ITS), with a load applied at a constant rate of deformation of (50 ± 2) mm/min. Finally, the test result (indirect tensile strength ratio, ITSR) was calculated according to the same European standard EN 12697-12:2008.

#### 2.2.3. Resistance to Permanent Deformation

The susceptibility of the studied mixtures to deformation was assessed by wheel tracking tests on a small-size device (OMADISA, Madrid, Spain), using Procedure B (in the air) and respecting the European standard EN 12697-22 [45]. For each mixture, two slabs with a volume of 30 × 30 × 4 cm^3^ were prepared and compacted with a vibratory compactor, respecting EN 12697-32 [41]. Each test was performed after 7 days of curing time.

The adopted test temperature was equal to 50 °C (EN 13108-20-reference D.1.5 [46]) and all the samples were conditioned at this constant temperature for a period of 4 h (prior to testing).

The rut depth formed on the slabs by repeated passing of a loaded wheel was measured. This load (700 N) was applied at a frequency of (26.5 ± 1.0) load cycles/min and the test ended when 10,000 load cycles were applied. The main parameters obtained in this test method are the wheel-tracking slope in the air (WTS_AIR_) and the mean rut depth in the air (RD_AIR_).

## 3. Results and Discussion

### 3.1. Aggregate–Bitumen Affinity Test

**Figure 3:**
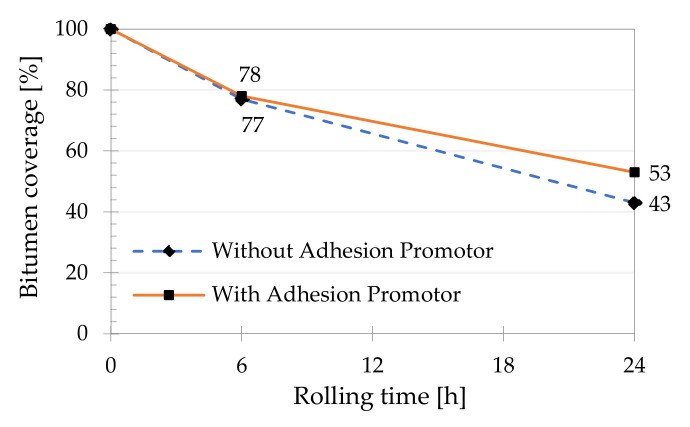
Bitumen coverage on uncompacted bitumen coated mineral aggregate particles.

The arithmetic average of the results obtained in the affinity tests is presented in Figure 3. It can be observed that the difference in the coating of the granitic aggregate after 6 h was residual in both tests, while a significant variation was found after 24 h.

As mentioned before, the difference in coating percentage after 6 h was only 1%. The uncertainty present in the subjective assessment of this value could be minimized using, for example, a hyperspectral imaging and digital picture analysis, as recently pointed out by Mei et al. [30].

**Figure 4:**
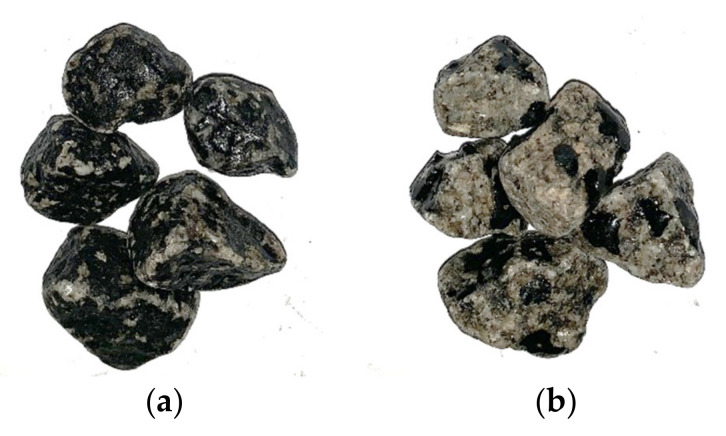
Granitic aggregates after affinity test (24 h): with (**a**) and without adhesion promoter (**b**).

However, after 24 h of testing, the mixture with the additive was higher by 23% (53 against 43%), confirming the effect of the adhesion promoter and the trend observed by Porot et al. [47], who concluded that the rolling bottle test begins to be truly differentiating after this period. In this case, the discrepancy in aggregates coating was already quite evident after 24 h of testing, as can be seen in the examples shown in Figure 4.

These results showed that the use of the anti-stripping agent led to similar conclusions to those reached by other researchers, namely, Liu et al. [23], Paliukaite et al. [48] and Lucas Júnior et al. [15].

According to one of these groups of researchers [48], the use of certain adhesion promoters, in a proportion of 0.4 wt.%, can increase the surface of the aggregates coated with bitumen more than 60% (after 6 h) and up to 73% (after 24 h). These authors came to this conclusion after evaluating the coating of granitic aggregates with two binders (50/70 and PMB 45/80-55) and two adhesion promoters, having tested 64 samples in total.

The obtained results can also be confirmed and correlated with the contact angles between binder and coarse aggregate, namely, through observations carried out with the OM and X-ray powder diffraction (XRPD), as did the researchers Caputo et al. [29].

### 3.2. Water Sensitivity Test

**Figure 5:**
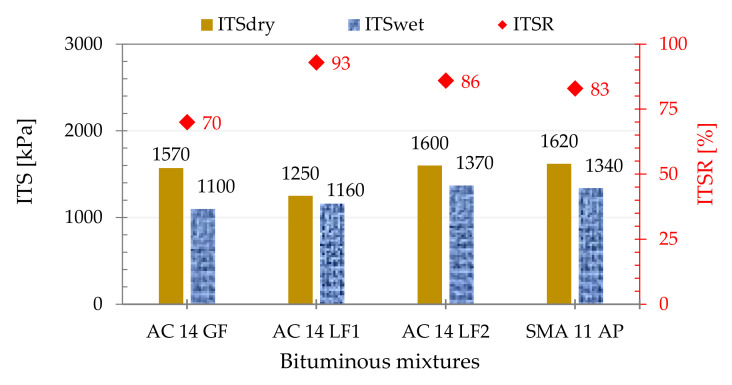
Water sensitivity test: ITS (average of three samples) (kPa) and ITSR (%).

Figure 5 presents the results of the water sensitivity test. As expected, dry specimens showed higher ITS values due to the effect of the presence of water on the porosity of the wet specimens.

The Portuguese Road Administration Specifications [49] do not refer to any requirement for the water sensitivity test, but the Spanish standard, PG3-3 [39], specify a minimum ITSR value of 80% for dense mixtures. The AC 14 *LF1/LF2* and SMA 11 *AP* mixtures obtained values ranging from 83 to 93%; therefore, all of them are above that reference value. As the European standard (EN 12697-12:2018) allows different energy levels in the impact compaction of these specimens (2 × 25, 2 × 35 or 2 × 50 blows), the results obtained, and the thresholds referred to in the technical specifications, have to be indexed to the number of blows adopted. This has been confirmed by several authors, namely, Wróbel et al. [50], who evaluated the reduction that takes place (under certain conditions) in the ITS of subcompacted asphalt mixtures, not only in the dry state, but also after conditioning in water.

The results in AC 14 *LF1* (ITSR = 93%) and AC 14 *LF2* (ITSR = 86%) confirmed the tendency described by different authors, namely, Choudhary et al. [9]: the increase in the amount of filler leads to a reduction in water resistance (active and passive bonds between aggregates and binder are reduced). The active adhesion is defined as the capacity of the binder to guarantee complete coverage of aggregates during the production of the bituminous mixture, while passive bonding is the ability of the binder to stay bonded to the aggregates throughout its service life. For this reason, Pasandín et al. [8] recommended that the selection of the filler must be performed carefully so that its introduction does not impair active or passive adhesion.

### 3.3. Resistance to Permanent Deformation

**Figure 6:**
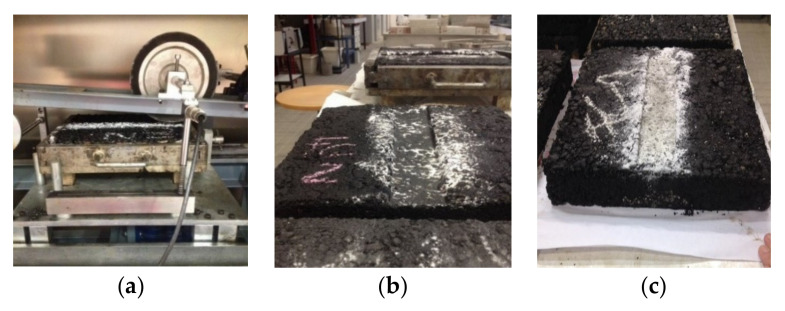
Permanent deformation test: wheel tracker (**a**) and some slabs after testing (**b**,**c**).

The wheel tracker used to assess permanent deformation resistance, as well as the appearance of some of the tested slabs, are shown in Figure 6.

**Figure 7:**
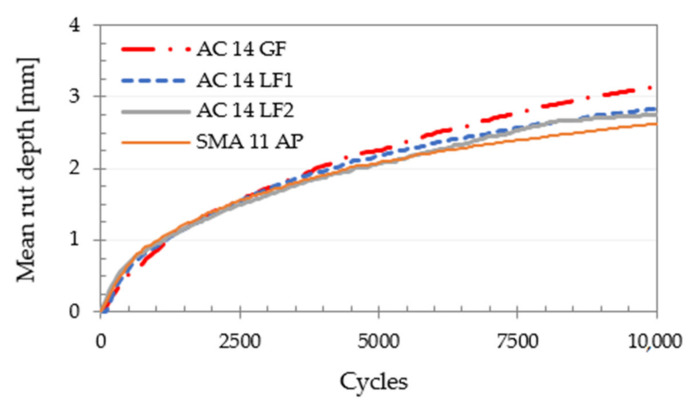
Resistance to permanent deformation: mean rut depth results (@ 50 °C).

Figure 7 shows the results on rut depth, depending on the number of cycles, for the studied bituminous mixtures. It is possible to observe that the effect of adding adhesion promoter (mixed with the cellulosic fibers) and the mineral skeleton of the SMA 11 *AP* led to a slight improvement in resistance to permanent deformation (@ 50 °C), even though this bituminous mixture has a higher binder content (unmodified) with a low softening point temperature (t_R&B_ = 47 °C).

The mean results of wheel tracking tests are presented in Table 4, taking into account the main parameters that characterize the permanent deformation resistance of the four bituminous mixtures assessed.

In fact, the AC 14 *LF1* and *LF2* and SMA 11 *AP* mixtures presented very similar average values. However, the SMA 11 *AP* mixture (with a discontinuous grading curve and including an adhesion promoter) showed the best results. The improvement of the resistance to permanent deformation for this mixture is noticeable, despite having a higher bitumen content (when compared to conventional AC mixtures). Furthermore, this increase in bitumen content (which led to a thicker film) will reduce aging, moisture damage [51] and also result in greater durability of the bituminous mixture, as mentioned by Maia [52] and Miranda et al. [53].

In this respect, the stiffness and fatigue resistance evaluation of these bituminous mixtures, as well as the study of other mixture types, before and after submitting to an aging procedure [such as rolling thin film oven (RTFO) + pressure aging vessel (PAV) or ultraviolet (UV) + infrared (IR) radiations alternated with water conditioning], may also be included in future research. The results in these parameters will complete the mechanical performance evaluation, allowing for more accurate indications about their life expectancy.

Further research can be oriented to confirm if these aggregates also present the same complex morphological characteristics that induce a better performance resistance (as concluded by [4,54]) or to deduct which parameters can be improved to optimize its morphology and the correspondent bituminous mixture behavior.

The use of the “locking point” concept, as suggested by Polaczyk et al. [55], can still be added to this study. This model was developed to replace the “Ndesing” standard (used in the Superpave mix design) to limit over compaction. These authors were able to demonstrate, through specific performance tests (Flow Number and IDEAL CT), the influence of “aggregate interlocking” on the phenomena of permanent deformation and fatigue failure.

Finally, an advanced binder characterization can also be carried out (in samples submitted to one of the known aging procedures), in order to better understand its rheologic behavior evolution over time and its correlation with the state of the interface with the aggregates. This assessment can be performed in samples of different ages using, for example, Fourier transform infrared spectroscopy (FTIR), dynamic mechanical analysis (DMA) and/or dynamic shear rheometry (DSR).

## 4. Conclusions

The performance of four bituminous mixtures, with and without limestone filler or adhesion promoter, was described in this paper. Mixtures that incorporated the limestone filler or the anti-stripping agent exhibited better results in the tests performed, proving that the use of these materials is beneficial, despite representing a slight increase in its final cost (estimated around 2–6 EUR per tonne of bituminous mixture).

Based on all tests carried out, it is possible to infer some conclusions:

- The affinity test between the aggregate and bitumen confirmed the effect of the adhesion promoter included in the fiber pellets, especially after 24 h of testing. The percentage of the area covered with bitumen was about 23% higher than that found in the mixture without adhesivity promoter.
- All the bituminous mixtures with limestone filler or adhesion promoter exhibited good resistance to moisture damage and their ITSR values were similar (ranging from 83 to 93%). These values are correlated with the energy level used in impact compaction and also with the parameters adopted in conditioning and testing.
- In general, results in permanent deformation resistance indicated a better behavior of mixtures with limestone filler or adhesion promoter. A slightly higher rut depth was observed in the mixture that included only granitic filler, AC 14 *GF* (about 20% higher than that observed in SMA 11 *AP*), despite having a lower binder content (5.1 wt.%).
- It was also confirmed that the mixture with the highest bitumen content (SMA 11 *AP*) presented the best WTS_AIR_ and RD_AIR_ values (0.11 mm/10^3^ cycles and 2.62 mm, respectively). Most likely, this fact was related to a better redistribution of mastic provided by the adhesion promoter carrier (cellulosic fibers). These pellets also prevent the binder from draining down (allowing its retention in the mixture) and promote a better coating of coarse aggregates.

In summary, this research confirmed the general trend that points to the addition of limestone filler or anti-stripping agent (in AC and SMA mixtures) as an effective measure for improving the adhesivity between aggregates and bitumen. Consequently, an increase in resistance to water damage and permanent deformation took place. Thus, such compositions provide these types of bituminous mixtures with better performance during their life in service, longer durability and greater safety for users.

## Figures and Tables

**Table 1:** Aggregates and fillers characteristics.

Aggregates/Properties	Standards	Gravel		Stone Dust	Filler	
		5/15 mm	2/4 mm	0/4 mm	Granitic	Limestone
Fines content, F [%]	EN 933-1	0.2	1.0	7.4	-	-
Density, *ρ*_b_ [Mg/m^3^]	EN 1097-6	2.60	2.60	2.50	2.63 ^(a)^	2.76 ^(a)^
Water absorption, WA_24_ [%]	EN 1097-6	0.6–0.9	<1	<1	-	-
Resist. to fragmentation, LA [%]	EN 1097-2	20	25	-	-	-
Flakiness Index, FI	EN 933-3	15	20	-	-	-
Rigden voids, V [%]	EN 1097-4	-	-	-	40.7	43.7
Fineness	EN 459-1	-	-	-	-	90 µm ≤ 15%
Ca(OH)_2_ content [%]	EN 459-1	-	-	-	-	≥4

^(a)^ Determined on a Helium pycnometer (AccuPyc 1330 Gas Pycnometer).

**Table 2:** Compositions of four bituminous mixtures studied (wt.%).

Materials/Mixtures/Contents (^a^)	AC 14 *GF*	AC 14 *LF1*	AC 14 *LF2*	SMA 11 *AP*
Bitumen 50/70	5.1	5.1	5.3	5.8
Limestone Filler, *LF*	-	1.8	4.7	7.5
Granitic Filler, *GF*	1.8	-	-	-
Stone dust 0/4 mm	39.7	39.6	16.1	15.1
Gravel 2/4 mm	16.3	16.4	21.8	30.1
Gravel 5/15 mm	37.1	37.1	52.1	41.2
Adhesion Promoter, *AP* (^b^)	-	-	-	0.3

(^a^) Percentages in the bituminous mixtures; (^b^) Included in cellulosic fiber pellets.

**Table 3:** Volumetric properties evaluated in each bituminous mixture.

Bituminous Mixtures	Bitumen [%]	Maximum Density [Mg/m^3^]	Air Voids [%]	VMA [%]	VFB [%]
AC 14 *GF*	5.1	2.38	3.4	14.7	77.2
AC 14 *LF1*	5.1	2.32	2.5	14.0	82.0
AC 14 *LF2*	5.3	2.40	4.0	15.9	74.7
SMA 11 *AP*	5.8	2.39	2.5	15.6	84.3

**Table 4:** Parameters evaluated in the permanent deformation tests (@ 50 °C).

Parameters	AC 14 *GF*	AC 14 *LF1*	AC 14 *LF2*	SMA11 *AP*
Wheel-tracking slope, WTS_AIR_ (mm/10^3^ cycles)	0.18	0.13	0.14	0.11
Mean rut depth, RD_AIR_ (mm)	3.16	2.84	2.76	2.62

## Data Availability

The data presented in this study are available on request from the corresponding author.

## Abbreviations

AAEM	Active Adhesion Evaluation Method
AC	Asphalt Concrete
AFM	Atomic Force Microscopy
BBS	Binder Bond Strength
DIP	Digital Image Processing
DMA	Dynamic Mechanical Analysis
DSR	Dynamic Shear Rheometry
EDS	Energy Disperse Spectroscopy
EDX	Energy Dispersive X-ray
FPC	Factory Production Control
FTIR	Fourier Transform Infrared Spectroscopy
HWT	Hamburg Wheel Tracker
IR	InfraRed spectroscopy or radiation
ITS	Indirect Tensile Strength
ITSR	Indirect Tensile Strength Ratio
MDS	Molecular Dynamics Simulation
OM	Optical Microscopy
PAV	Pressure Aging Vessel
RBT	Rolling Bottle Test
RD	Rut Depth
rpm	Revolutions per minute
RTFO	Rolling Thin Film Oven
SCA	Silane Coupling Agent
SEM	Scanning Electron Microscopy
SLD	Simplex Lattice Design
SMA	Stone Mastic Asphalt
t_R&B_	Ring and ball temperature
UV	UltraViolet radiation
WMA	Warm Mix Asphalt
WTS	Wheel-Tracking Slope
WTT	Wheel Tracking Test
XPS	X-ray Photoelectron Spectroscopy
XRPD	X-ray powder diffraction

## References

[1] Zhang G., Qiu J., Zhao J., Wei D., Wang H. (2020). Development of Interfacial Adhesive Property by Novel Anti-Stripping Composite between Acidic Aggregate and Asphalt. Polymers.

[2] Mehrara A., Khodaii A. (2013). A review of state of the art on stripping phenomenon in asphalt concrete. Constr. Build. Mater..

[3] Aguiar-Moya J.P., Baldi-Sevilla A., Salazar-Delgado J., Pacheco-Fallas J.F., Loria-Salazar L., Reyes-Lizcano F., Cely-Leal N. (2018). Adhesive properties of asphalts and aggregates in tropical climates. Int. J. Pavement Eng..

[4] Wang W., Cheng Y., Tan G., Tao J. (2018). Analysis of Aggregate Morphological Characteristics for Viscoelastic Properties of Asphalt Mixes Using Simplex Lattice Design. Materials.

[5] Kassem E., Garcia Cucalon L., Masad E., Little D. (2018). Effect of warm mix additives on the interfacial bonding characteristics of asphalt binders. Int. J. Pavement Eng..

[6] Lesueur D., Petit J., Ritter H.-J. (2012). The mechanisms of hydrated lime modification of asphalt mixtures: A state-of-the-art review. Road Mater. Pavement Des..

[7] Alvarez A.E., Ovalles E., Caro S. (2012). Assessment of the effect of mineral filler on asphalt-aggregate interfaces based on thermodynamic properties. Constr. Build. Mater..

[8] Pasandín A.R., Pérez I. (2015). The influence of the mineral filler on the adhesion between aggregates and bitumen. Int. J. Adhes. Adhes..

[9] Choudhary J., Kumar B., Gupta A. (2020). Effect of filler on the bitumen-aggregate adhesion in asphalt mix. Int. J. Pavement Eng..

[10] Cui P., Wu S., Xiao Y., Wang F., Wang F. (2019). Quantitative evaluation of active based adhesion in Aggregate-Asphalt by digital image analysis. J. Adhes. Sci. Technol..

[11] Martinho F.C.G., Farinha J.P.S. (2019). An overview of the use of nanoclay modified bitumen in asphalt mixtures for enhanced flexible pavement performances. Road Mater. Pavement Des..

[12] Hamedi G.H., Sahraei A., Esmaeeli M.R. (2021). Investigate the effect of using polymeric anti-stripping additives on moisture damage of hot mix asphalt. Eur. J. Environ. Civ. Eng..

[13] Siddiqui M.N., Baig M.G., Ali M.F. (2009). Effect of Chemical Additives on the Binding Strength of Arabian Asphalts. Pet. Sci. Technol..

[14] Cui S., Blackman B.R.K., Kinloch A.J., Taylor A.C. (2014). Durability of asphalt mixtures: Effect of aggregate type and adhesion promoters. Int. J. Adhes. Adhes..

[15] Lucas Júnior J.L.O., Babadopulos L.F.A.L., Soares J.B., Souza L.T. (2021). Evaluating the effect of amine-based anti-stripping agent on the fatigue life of asphalt pavements. Int. J. Pavement Eng..

[16] Ding G., Yu X., Dong F., Ji Z., Wang J. (2020). Using Silane Coupling Agent Coating on Acidic Aggregate Surfaces to Enhance the Adhesion between Asphalt and Aggregate: A Molecular Dynamics Simulation. Materials.

[17] Liu Z., Huang X., Sha A., Wang H., Chen J., Li C. (2019). Improvement of Asphalt-Aggregate Adhesion Using Plant Ash Byproduct. Materials.

[18] Zhang J., Apeagyei A.K., Airey G.D., Grenfell J.R.A. (2015). Influence of aggregate mineralogical composition on water resistance of aggregate–bitumen adhesion. Int. J. Adhes. Adhes..

[19] Yin Y., Chen H., Kuang D., Song L., Wang L. (2017). Effect of chemical composition of aggregate on interfacial adhesion property between aggregate and asphalt. Constr. Build. Mater..

[20] Feng P., Wang H., Ding H., Xiao J., Hassan M. (2020). Effects of surface texture and its mineral composition on interfacial behavior between asphalt binder and coarse aggregate. Constr. Build. Mater..

[21] Fan Z., Lin J., Chen Z., Liu P., Wang D., Oeser M. (2021). Multiscale understanding of interfacial behavior between bitumen and aggregate: From the aggregate mineralogical genome aspect. Constr. Build. Mater..

[22] Cong P., Guo X., Ge W. (2021). Effects of moisture on the bonding performance of asphalt-aggregate system. Constr. Build. Mater..

[23] Liu Y., Apeagyei A., Ahmad N., Grenfell J., Airey G. (2014). Examination of moisture sensitivity of aggregate-bitumen bonding strength using loose asphalt mixture and physico-chemical surface energy property tests. Int. J. Pavement Eng..

[24] Zhang X., Wang J., Zhou X., Zhang Z., Chen X. (2021). Mechanical Properties of the Interfacial Bond between Asphalt-Binder and Aggregates under Different Aging Conditions. Materials.

[25] Horgnies M., Darque-Ceretti E., Fezai H., Felder E. (2011). Influence of the interfacial composition on the adhesion between aggregates and bitumen: Investigations by EDX, XPS and peel tests. Int. J. Adhes. Adhes..

[26] Lv X., Fan W., Wang J., Liang M., Qian C., Luo H., Nan G., Yao B., Zhao P. (2019). Study on adhesion of asphalt using AFM tip modified with mineral particles. Constr. Build. Mater..

[27] Ji X., Li J., Zhai X., Zou H., Chen B. (2020). Application of Atomic Force Microscope to Investigate the Surface Micro-Adhesion Properties of Asphalt. Materials.

[28] Khasawneh M.A., Al-Oqaily D.M., Abu Alia A.H., Al-Omari A.A. (2021). Evaluation of aggregate-binder bond strength using the BBS device for different road materials and conditions. Int. J. Pavement Eng..

[29] Caputo P., Miriello D., Bloise A., Baldino N., Mileti O., Ranieri G.A. (2020). A comparison and correlation between bitumen adhesion evaluation test methods, boiling and contact angle tests. Int. J. Adhes. Adhes..

[30] Mei A., Fusco R., Moroni M., Fiore N., Fontinovo G., D’Andrea A. (2021). Affinity between Bitumen and Aggregate in Hot Mix Asphalt by Hyperspectral Imaging and Digital Picture Analysis. Coatings.

[31] Javilla B., Fang H., Mo L., Shu B., Wu S. (2017). Durability of Innovative Construction Materials and Structures Test evaluation of rutting performance indicators of asphalt mixtures. Constr. Build. Mater..

[32] Afonso M.L., Dinis-Almeida M., Fael C.S. (2017). Study of the porous asphalt performance with cellulosic fibres. Constr. Build. Mater..

[33] Han J., Shiwakoti H. (2016). Wheel tracking methods to evaluate moisture sensitivity of hot-mix asphalt mixtures. Front. Struct. Civ. Eng..

[34] CEN—European Committee for Standardization (2020). European Standard EN 12697-34. Bituminous Mixtures—Test Methods—Part 34: Marshall Test.

[35] CEN—European Committee for Standardization (2017). European Standard EN 1426. Bitumen and Bituminous Binders; Determination of Needle Penetration.

[36] CEN—European Committee for Standardization (2017). European Standard EN 1427. Bitumen and Bituminous Binders; Determination of the Softening Point; Ring and Ball Method.

[37] CEN—European Committee for Standardization (2016). European Standard EN 13108-1. Bituminous Mixtures—Material Specifications—Part 1: Asphalt Concrete.

[38] Martinho F., Lanchas S., Nunez R., Batista F., Miranda H. The Portuguese experience in SMA-type bituminous mixtures with cellulosic fibers. Proceedings of the 7th Congresso Rodoviário Português.

[39] Dirección General de Carreteras (2014). General Technical Specifications for Road and Bridge Works, PG-3.

[40] CEN—European Committee for Standardization (2018). European Standard EN 12697-30. Bituminous Mixtures—Test Methods for Hot Mix Asphalt—Part 30: Specimen Preparation by Impact Compactor.

[41] CEN—European Committee for Standardization (2019). European Standard EN 12697-32. Bituminous Mixtures—Test Methods for Hot Mix Asphalt—Part 32: Laboratory Compaction of Bituminous Mixtures by Vibratory Compactor.

[42] CEN—European Committee for Standardization (2020). European Standard EN 12697-11. Bituminous Mixtures—Test Methods—Part 11: Determination of the Affinity Between Aggregate and Bitumen.

[43] CEN—European Committee for Standardization (2018). European Standard EN 12697-12. Bituminous Mixtures—Test Methods—Part 12: Determination of the Water Sensitivity of Bituminous Specimens.

[44] CEN—European Committee for Standardization (2017). European Standard EN 12697-23. Bituminous Mixtures—Test Methods—Part 23: Determination of the Indirect Tensile Strength of Bituminous Specimens.

[45] CEN—European Committee for Standardization (2020). European Standard EN 12697-22. Bituminous Mixtures—Test Methods—Part 22: Wheel Tracking.

[46] CEN—European Committee for Standardization (2016). European Standard EN 13108-20. Bituminous Mixtures—Material Specifications—Part 20: Type Testing.

[47] Porot L., Soenen H., Apeagyei A., Grenfell J., Vansteenkiste S., Chailleux E. (2018). Recommendation of RILEM TC 237-SIB on affinity between aggregates and bituminous binders. Mater. Struct..

[48] Paliukaitė M., Vorobjovas V., Bulevičius M., Andrejevas V. (2016). Evaluation of different test methods for bitumen adhesion properties. Transp. Res. Procedia.

[49] Infrastruturas de Portugal, SA (2014). Work-Type Specifications (CETO), 14.03—Paving Characteristics of Materials. https://servicos.infraestruturasdeportugal.pt/pdfs/infraestruturas/14_03_set_2014.pdf.

[50] Wróbel M., Woszuk A., Franus W. (2020). Laboratory Methods for Assessing the Influence of Improper Asphalt Mix Compaction on Its Performance. Materials.

[51] Radhakrishnan V., Ramya Sri M., Reddy K.S. (2020). Sensitivity of rutting and moisture resistance of asphalt mixes to gradation and design air void content. Int. J. Pavement Eng..

[52] Maia M. (2018). Influence of Aggregate/Bitumen Adhesivity on the Performance of a Bituminous Mixture. Master’s Thesis.

[53] Miranda H.B., Batista F.A., Neves J., de Lurdes Antunes M. (2020). Influence of the aggregate skeleton matrix and volumetric composition on the resistance of stone mastic asphalt to permanent deformation. Road Mater. Pavement Des..

[54] Cheng Y., Wang W., Tao J., Xu M., Xu X., Ma G., Wang S. (2018). Influence Analysis and Optimization for Aggregate Morphological Characteristics on High- and Low-Temperature Viscoelasticity of Asphalt Mixtures. Materials.

[55] Polaczyk P., Ma Y., Xiao R., Hu W., Jiang X., Huang B. (2021). Characterization of aggregate interlocking in hot mix asphalt by mechanistic performance tests. Road Mater. Pavement Des..

